# *OGDHL* Variant rs2293239: A Potential Genetic Driver of Chinese Familial Depressive Disorder

**DOI:** 10.3389/fpsyt.2022.771950

**Published:** 2022-03-18

**Authors:** Zhi Pan, Hongjun Tian, Tao Fang, Zhidong Liu, Xiangdong Liu, Guangqian Dou, Guoyong Huang, Zhenqing Zhang, Guangdong Chen, Wenqiang Wang, Chuanjun Zhuo

**Affiliations:** ^1^Key Laboratory of Genetic Psychiatry, Wenzhou Seventh People Hospital, Wenzhou, China; ^2^Key Laboratory of Real Time Tracing of Brain Circuits of Neurology and Psychiatry, Nankai University Affiliated Tianjin Fourth Center Hospital, Tianjin Medical University Affiliated Tianjin Fourth Center Hospital, Tianjin Fourth Center Hospital, Tianjin, China; ^3^Department of Psychiatry, Xiamen Xianyue Hospital, Xiamen, China; ^4^Department of Psychiatry, The First Affiliated Hospital of Zhengzhou University, Zhengzhou, China; ^5^Biological Psychiatry International Joint Laboratory of Henan, Zhengzhou University, Zhengzhou, China

**Keywords:** *OGDHL*, rs2293239, depressive disorder, Chinese, genetic driver

## Abstract

Depressive disorders are a severe psychiatric and social problem that affect more than 4% of the global population. Depressive disorders have explicit hereditary characteristics; however, the precise driving genetic force behind these disorders has not yet been clearly illustrated. In the present study, we recruited a three-generation Chinese pedigree in which 5 of 17 members had long-term depression. We conducted whole-exome sequencing to identify the genetic mutation profiles of the family, and a list of susceptible genetic variations that were highly associated with depression onset was revealed via multiple omics analysis. In particular, a non-synonymous single nucleotide variation in the oxoglutarate dehydrogenase-like (*OGDHL*) gene, rs2293239 (p.Asn725Ser), was identified as one of the major driving genetic forces for depression onset in the family. This variant causes an important conformational change in the transketolase domain of *OGDHL*, thus reducing its binding affinity with the cofactor thiamine pyrophosphate and eventually resulting in the abnormal accumulation of glutamate in the brain. Brain imaging analysis further linked the rs2293239 variant with an enlarged amygdala and cerebellum in depressive family members. In summary, the present study enhances the current genetic understanding of depressive disorders. It also provides new options for prioritizing better clinical therapeutic regimens, as well as identifying a new protein target for the design of highly specific drugs to treat depressive disorders.

## Introduction

Depressive disorders, or depression, refers to common but severe mental disorders that affect more than 264 million people worldwide (1). They are also a leading cause of disability, and place heavy economic burdens on both patients' families and society (2). Unfortunately, clinical therapy for depressive disorders, and in particular major depressive disorder (MDD), usually results in poor outcomes because of the heterogeneous pathophysiology of these disorders. Any one or a combination of biological, physical, genetic, and social factors may account for depression onset. Although great efforts have been made over many years, the driving force underlying depressive disorders remains unclear in most cases, which has little benefit for improving therapeutic regimens (3, 4).

Depressive disorders are heritable. Early twin studies estimated the heritability of depression to be about 36% (5, 6). For MDD, heritability was determined to be approximately 32% in a study that measured genomic similarity among unrelated individuals (7). The inheritance of depression from generation to generation makes it feasible to reveal the potential driving genetic force of this disorder. For example, Hu conducted comparative genotyping of a case–control cohort and reported that a single nucleotide polymorphism, rs25531 in *SLC6A4*, likely impacts depression onset by interfering with the serotonin pathway (8). In addition, Zubenko et al. performed a linkage analysis on 81 families and identified 19 loci that were suspected to relate to depressive disorders (9). Camp et al. reported three loci related to depression or anxiety based on 87 Utah pedigrees (10). Furthermore, Kendler and Flint summarized a number of genes (*5HTTP*/*SLC6A4, APOE, DRD4, GNB3, HTR1A, MTHFR*, and *SLC6A3*) linked to susceptibility to heterogeneous depression by interpreting a large amount of candidate gene literature in 2014 (11). However, despite convincing evidence for their genetic contribution to disease susceptibility, only a few of these genes or loci have substantial molecular evidence to support the diagnoses of the members of this family and clinical therapy (11, 12). In recent years, genome-wide association studies (GWAS) have been applied to the search for genetic associations in various psychiatric diseases. Several biomarker gene variants have been proposed for depression in different ethnic populations, including *BICC1* rs9416742 in a UK population (13), *SIRT1* rs12415800 in a Chinese Han population (14), and rs12462886 of a non-coding region in a US population (15). However, the results of GWAS for MDD have been questioned because of a lack of generalizability and interpretability (11). It has also been proposed that previously identified candidate genes are likely to be false positives (16). In particular, the variants identified using GWAS approaches usually have low penetrance, and few have had their associations with depression onset confirmed molecularly. To date, strong depression-associated variants have not yet been identified; a large gap remains for the translation of our current knowledge of depressive disorders to efficient clinical therapy.

To narrow this gap, in the present study, we conducted a pedigree analysis in a three-generation Chinese family with depression. We first portrayed the genetic mutation profiles for every family member, from which we identified rare variants with high penetrance that were associated with familial depression. We then performed a protein structure–activity relationship analysis and multimodal brain image analyses to interpret the potential roles of the selected variants in depression onset.

## Materials and Methods

This study was approved by the Clinical Research Ethics Committee of the Xiamen Xianyue Hospital, Fujian, China. All experimental protocols were performed in accordance with the Declaration of Helsinki. Written informed consent was obtained from all participants after a complete description of the study.

### Family Pedigree

**Figure 1:**
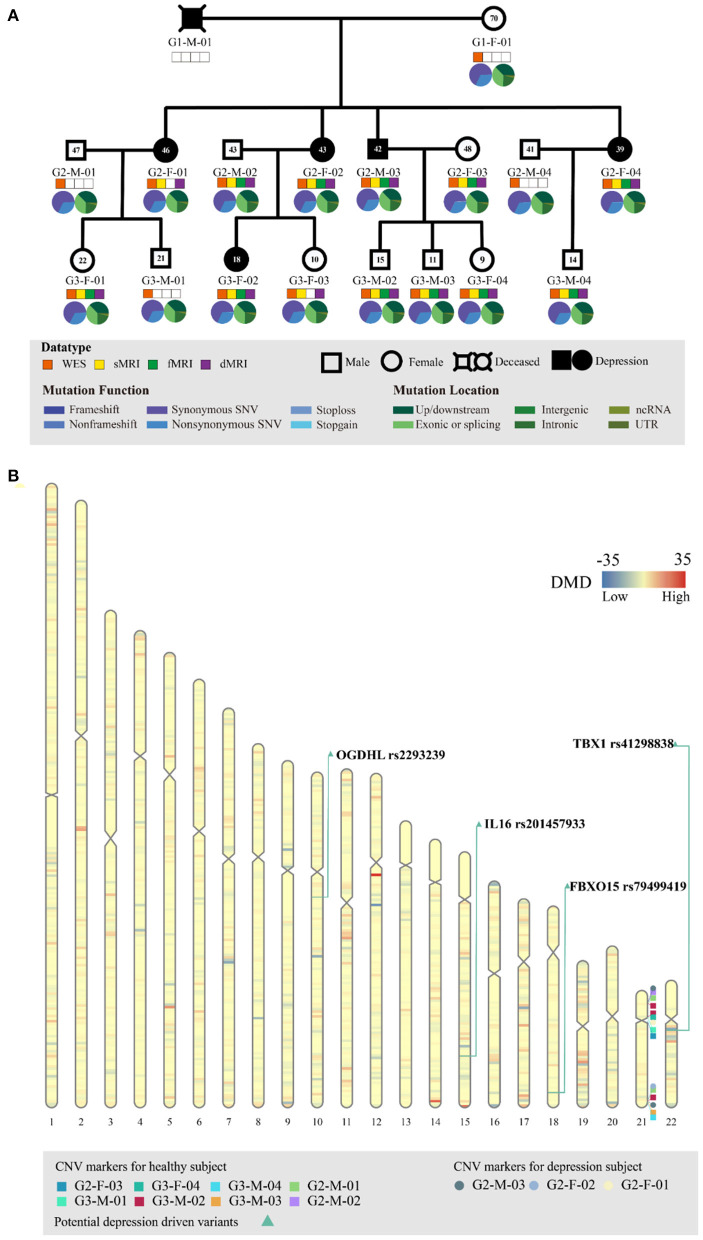
Genetic profiling of the depressive family. **(A)** The pedigree structure and their genetic mutation profiles. The number inside each large circle or rectangle indicates the age of the family member. The small colored rectangles under the family members indicate the type of data acquired in this study. The pie chart illustrates the mutation composition. **(B)** The differential mutation density (DMD) landscapes in chromosomes. Several hotspots of differential variants, at 12q12 (DMD = 32.9833), 14q32.33 (DMD = 24.5667), and 15q26.3 (DMD = 22.3000), were observed in the depressive family members, covering 19 genes and 976 variants.

The pedigree in this study was a three-generation family of 17 members, five of whom were diagnosed with different levels of depressive disorders by multiple doctors from the Xiamen Xianyue Hospital. Diagnoses were made strictly according to the guidelines of the International Classification of Diseases, Tenth Revision (ICD-10), and pathophysiology was carefully assessed by reviewing a thorough history, examination, and workup of each patient. The pedigree at the time of this study is illustrated in Figure 1 and demographic information is shown in Table 1.

**Table 1:** Demographic and clinical information of the depressive subjects.

	**G2-F-01**	**G2-F-04**	**G2-F-03**	**G2-F-02**	**G3-F-02**
Age	46	39	41	43	18
Sex	Female	Female	Male	Female	Female
Depression grade	Mild to moderate depression	Major depression	Major depression	Major depression	Mild to moderate depression
HDRS-17 score	22	42	34	33	20
Duration of illness (months)	148	187	67	184	17
Medication	Paroxetine hydrochloride	Lamotrigine olanzapine	Fluoxetine hydrochloride	Lamotrigine fluvoxamine	Lamotrigine fluvoxamine
Duration of treatment (months)	102	138	56	132	16

*Data were calculated up to 2018*.

*HDRS-17, hamilton depression rating scale*.

For each family member, 1 mL of peripheral blood was collected in the hospital. Subsequently, plasma separation was conducted by centrifugation at 800 × g for 10 min and 2,500 × g for 10 min consecutively. The separated leukocytes were stored at −80°C before the DNA was extracted.

### Mutation Profiling by Whole-Exome Sequencing

The genomic DNA of leukocyte samples was extracted using the EZ-10 Spin Column Blood Genomic DNA Purification Kit (Sangon Biotech Co, Ltd., Shanghai, China). The DNA concentration was measured using the Qubit^®^ DNA Assay Kit and Qubit^®^ 2.0 Fluorometer (Life Technologies, CA, USA). For each sample, 0.6 μg of DNA was used as the input material for the DNA sample preparation. The whole exome was captured using the Agilent SureSelect Human All Exon Kit (Agilent Technologies, CA, USA) and the library for sequencing was prepared according to the manufacturer's instructions. WES was performed by Novogene (Beijing, China) using the Illumina Hiseq platform in a 150-base pair (bp) paired-end mode.

### Exome Data Preprocessing and Variant Calling

Before variant calling, quality control was conducted on the raw sequencing data using Trimmomatic (version 0.36; parameters: LEADING = 3, TRAILING = 3, SLIDINGWINDOW = 4:15, MINLEN = 50, http://www.usadellab.org/cms/?page=trimmomatic) (17). Subsequently, Kraken (18) was used to exclude potentially polluted reads. The clean reads were then mapped to the human reference genome (GRCh38) using the Burrows–Wheeler Aligner (19) (v.0.7.17; parameters: mem -t 4 -M). We then used the Genome Analysis Toolkit (20) (GATK, v.4.0.10.1) and Samtools (21) (v.1.9) for basic processing, duplicate marking, and base quality score recalibration. Next, variant calling for germline mutations was conducted using the GATK HaplotypeCaller. Variants were further annotated using ANNOVAR (22) (v.2018Apr16) by referring to databases such as refGene, avsnp150, clinvar_20180603, dbnsfp35a, exac03, exac03nontcga, exac03nonpsych, cosmic70, 1000g2015aug_all, and 1000g2015aug_eas.

### Differential Mutation Density

The DMD was determined by summarizing the mutation difference between the depressive and healthy members of the family for every 1,000,000 bp (1 Mbp) segment of chromosome:


(1) $$\begin{array}{r} DMD=\sum_{i=1}^{n}\left(\frac{O_{id}}{N_{d}}\;-\;\frac{O_{i,h}}{N_{h}}\right) \end{array}$$

where O_i, *d*_ indicates the occurrence of a definite mutation *i* in the depressive members, N_*d*_ indicates the number of depressive family members, O_i, *h*_ indicates the occurrence of a definite mutation *i* in the healthy family members, N_*h*_ indicates the number of depressive family members, and *n* indicates the total number of mutations detected in the 1 Mbp segment in this study.

### Copy Number Variation Calling and Differential Analysis

eXome Hidden Markov model (XHMM) (23) (v.1.0) and GATK (20) (v.4.0.10.1) were used separately to call CNVs based on exomes. The XHMM method accepted the Burrows–Wheeler Aligner-aligned BAM file as the input. The XHMM module (- PCA) was applied to generate component variation, other modules (- normalization and - matrix) were applied to generate a z-score of read depth and normalization, and the hidden Markov model (- discover) was applied to identify CNVs. The GATK method used the Determine Germline Contig Ploidy module to determine autosomal and allosomal contig ploidy, and subsequently used the Germline CNV Caller module to call CNVs. In the current study, only consensus CNVs that were called by both tools were retained for differential analysis. The CNV differential analysis was conducted between the depressive group and the healthy group using a self-written shell script. The differential CNVs, if available, were further functionally annotated to evaluate their connection with depression using Classify CNV (24) software (v.1.1.0) and referring to the human genome (GRCh38).

### Identification of Depression-Associated Variants

**Figure 2:**
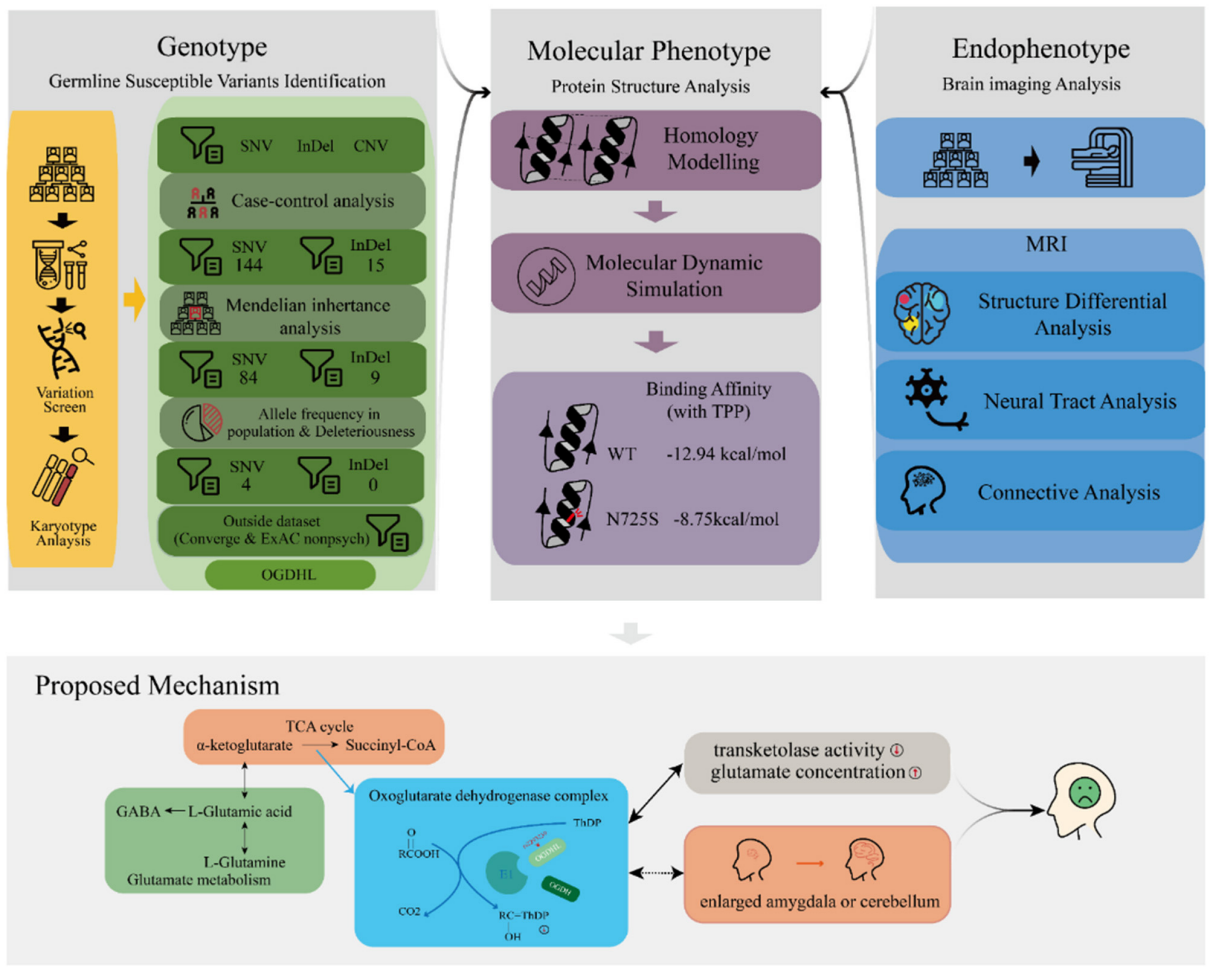
Schema of the discovery of driver variants for the familial depressive disorder.

We performed a series of bioinformatic analyses one by one to identify depression-associated variants (Figure 2), as follows. (1) A comparison of mutation profiles was performed to extract the differential, non-synonymous, and exonic variants. At the time of this investigation, the third generation of the family were mostly teenagers, when depression is likely under progression but symptoms have not yet appeared. Thus, members of the third generation were excluded from the case–control comparative analysis. The third-generation member G3-F-02, who was diagnosed with depression just before the initiation of this study, was the only exception; she was involved in the variant detection. (2) Genetic segregation analysis was conducted to confirm whether the variants satisfied a Mendelian inheritance model using the segreg program in the Statistical Analysis for Genetic Epidemiology package (25) (v.6.4). Sporadic variants that did not fit the Mendelian inheritance model were excluded because they did not contribute much to familial trait inheritance statistically according to the program. (3) Common variants, with an allele frequency of more than 0.05 in the Exome Aggregation Consortium (ExAC) (26) and the 1000 genome project (27, 28), were excluded. Odds ratios (ORs) were calculated for every retained rare variant following conventional practices, based on the allele frequency difference between the psychosis (ExAC_Psych) and non-psychosis (ExAC_nonPsych) subsets of ExAC release 1.0 (GRCh38). In the same way, we also calculated the ORs for every variant by counting the allele frequency difference between MDDs in Chinese Han women and normal controls in the Convergence of Epicardial and Endocardial Ablation for the Treatment of Symptomatic Persistent Atrial Fibrillation (CONVERGE) study (14). (4) For the remaining variants, deleteriousness was evaluated using 13 different algorithms in dbNSFP (29), including SIFT (30), PolyPhen2 HDIV, PolyPhen2 HVAR (31), LRT (32), MutationTaster (33), MutationAssessor (34), FATHMM (35), PROVEAN (36), MetaSVM, MetaLR (37), GERP++ (38), and PhyloP (39). The variants that were predicted deleterious by at least one algorithm were considered to have high penetrance for depression. Variants with more hits were considered to be more deleterious. The most deleterious variants were considered to be driving variants for depression and were adopted for later functional analyses.

### Protein Structure–Activity Relationship Analysis

Because the human OGDHL protein structure was not yet available in the Protein Data Bank (PDB) database, we used homology modeling to simulate the structure for the structure–activity relationship analysis. The simulation was conducted using the commercial software Molecular Operating Environment (MOE), adopting the multifunctional 2-oxoglutarate metabolism enzyme (PDBID: 2yic) as the template coupled with the cofactor thiamine pyrophosphate (TPP), Ca^2+^, and Mg^2+^. The simulated protein structure was stabilized and optimized in a water environment (310 K temperature) using GROMACS software (40) (v.2018.4) for 20 ns molecular dynamics with both force fields: amber general force field (41) and amber ff99SB-ILDN (42). Furthermore, we simulated the mutant protein (OGDHL N725S) by changing the amino acid at position 725 (NP_001334748.1) from asparagine (N) to serine (S), followed by 50 ns molecular dynamics under the same conditions as for the wild-type protein simulation. The structure–activity relationship analysis was performed by evaluating the conformational change and binding affinity between the OGDHL wild type and N725S mutant using MOE software.

### Image Data Acquisition and Processing

Magnetic resonance imaging (MRI) of the brain, which included structural MRI (sMRI), functional MRI (fMRI), and diffusion imaging (dMRI), were scanned for every family member when possible. For various reasons, all three imaging modalities were not used for all family members. The image data acquisition statuses are shown in Figure 1A. All images were taken using a MAGNETOM Skyra 3T MRI (Siemens AG, Erlangen, Germany). High-resolution T1-weighted sMRI images were acquired according to a standardized protocol (three-dimensional magnetization-prepared rapid acquisition with gradient echo sequence; repetition time [TR]/inversion time [TI]/echo time [TE] = 2,530/950/2.01 ms; 192 sagittal slices; flip angle = 8°; slice thickness = 1 mm; field of view = 256 × 256 mm^2^; base resolution = 256). The functional images were obtained using blood oxygen level-dependent contrast-sensitive gradient-echo planar imaging (TE = 30 ms; flip angle = 90°; in-plane resolution = 3.238 × 3.438 mm; volume TR = 2.1 s). The dMRI was acquired using echo-planar imaging (b = 1,000 s/mm^2^; TR = 11,700 ms; TE = 79 ms; 2 mm thickness, image matrix 112 × 112; field of view = 224 × 224 mm^2^).

The raw brain images were converted into NIfTI format using dcm2niix (43) (v.25) and were then output for external analyses. For the sMRI images, we used the FIRST module (44) (run_first_all) in FMRIB Software Library (FSL) software (45) (v.6.0.1) to extract the subcortical structure segmentation. Overall, 15 subcortical segments (left-thalamus, left-caudate, left-putamen, left-pallidum, left-hippocampus, left-amygdala, left-accumbens-area, brain-stem, right-thalamus-proper, right-caudate, right-putamen, right-pallidum, right-hippocampus, right-amygdala, and right-accumbens-area) and a cerebellum segment were extracted in this study. The volumes for the subcortical structures and cerebellum were determined using the “fslmath” program of FSL. The volume comparison between the healthy control and depression groups was performed using a randomization test in a self-written R script; its robustness was evaluated using the randomization test. For the fMRI images, raw images were processed for motion correction, field unwarping, normalization, and bias field correction using fMRIPrep (46) software. In the normalized images, the blood oxygen levels of 39 distinct brain regions (nodes) were determined using the Python package nilearn (47), referring to the multi-subject dictionary learning (MSDL) atlas (48). For each family member, the covariance values of the blood oxygen levels between the nodes were calculated to form a 39 × 39 connection matrix. The connectome map was built upon the connection matrix by setting an edge_threshold = 0.99, in which the edge stood for the connectivity [calculated by the tangent (49)] between the nodes. The connection map difference between the healthy control and depression groups was determined using the FSL module randomize (50). The dMRI images were preprocessed using the tract-based spatial statistics (TBSS) module (51) of FSL software to create the fractional anisotropy (FA) image, register the FA image, skeletonize the mean FA image, and project the FA information onto the mean FA skeleton image. The difference in the fiber tract skeleton between the healthy control and depressive groups was then evaluated using the FSL module randomize (50).

## Results

### Identification of Potential Driver Variants in Familial Depression

WES of the peripheral blood of all 17 family members yielded a mean sequencing depth of 258.94 × (ranging from 234.26 × to 294.52 ×) after quality control (Supplementary Table S1). The sequencing coverage was deep enough for robust variant calling. Overall, 141,022 single nucleotide variants (SNVs) and 19,148 insertion/deletion variants (InDel) were obtained. The distribution of SNVs and InDels in each family member is illustrated in Figure 1A. The family members had almost the same distributions of the different variant types, indicating that the variant calling from WES was well processed.

Before searching for depression-associated variants, we first examined chromosomal integrity by measuring the CNVs caused by abnormal chromosomal events, such as duplication, deletion, rearrangement, or recombination. There were no significant chromosomal differences between the depressive and healthy family members. In particular, no significant CNV differences were observed in the chromosomal region 22q11.2; this region has been linked with schizophrenia and other neuropsychiatric/behavioral disorders. Furthermore, we analyzed the DMD landscapes per 1 Mbp segment of chromosomes (Figure 1B). Three hotspots of DMD (with DMD > 20) were detected, at 12q12 (DMD = 32.9833), 14q32.33 (DMD = 24.5667), and 15q26.3 (DMD = 22.3000). These three DMD hotspots may be chromosomal fragments that are mutual to the depressive members, and are inherited from generation to generation (Figure 1B). These chromosomal fragments covered 19 genes and 976 variants: four genes in 12q12 (*SLC2A13, LRRK2, MUC19*, and *CNTN1*), five genes in 14q32.33 (*TDRD9, RASPG, KIF26A, C14orf180*, and *TMEM179*), and 10 genes in 15q26.3 (*LRRK, CHSY1, SELENOS, SNRPA1, PCSK6, TM2D3, TARS3, OR4F6, OR4F15*, and *OR4F4*). Although a literature search of these genes and variants revealed some clues of their association with depressive or other affective disorders, a series of bioinformatic analyses (including OR association analyses and deleteriousness analyses) on these variants did not confirm their significant association with or penetrance for depression in our pedigree. Hence, we no longer considered the variants at these depression-specific hotspots as the candidate driving force of familial depressive disorders.

To identify potential depression-associated variants, we conducted a series of bioinformatic analyses. The case–control analysis extracted 159 differential, non-synonymous, and exonic variants between the depressive and other family members, including 144 SNVs and 15 InDels. According to the segregation analysis based on the whole mutation profiles, the familial depression likely fit either an autosomal dominant or a recessive Mendelian inheritance gene model (Table 2). Hence, variants that did not fit Mendelian inheritance were excluded; 93 variants were consequently retained, including 84 SNVs and nine InDels. Furthermore, because the estimated incidence rate of depression worldwide is about 2–6% (52), driver genetic variants are more likely to be rare variants than common variants. Accordingly, common variants (with an allele frequency > 0.05 in the population) were also removed from the candidate list by referring to ExAC and the 1000 Genome project. Moreover, a deleteriousness analysis was conducted to evaluate the penetrance of the variants on depression. Only four SNV variants were considered to have high penetrance for depression: *OGDHL* rs2293239, *TBX1* rs41298838, *IL16* rs201457933, and *FBXO15* rs79499419 (Table 3).

**Table 2:** Segregation analysis of the family based on the whole-exome sequencing data.

**Mode of inheritance**	**LN (likelihood)**	**-2LN (Lh)**	**AIC**
Sporadic model without familiar residual association	−8.3785	16.757	26.757
Sporadic model with familiar residual association	−6.75353	13.507	23.5071
Mendelian major gene models with autosomal dominant inheritance	−7.66576	15.3315	23.2215
Mendelian major gene models with autosomal recessive inheritance	−7.66575	15.332	23.2216

**Table 3:** Information and allele frequency for the four potential driving variants.

**SNP**	**rs2293239**	**rs201457933**	**rs79499419**	**rs41298838**
Chromosome	chr10	chr15	chr18	chr22
Start	49739806	81300079	74126069	19765921
End	T	G	T	G
Reference	C	C	C	A
Gene.refGene	OGDHL	IL16	FBXO15	TBX1
ExAC_EAS/ExAC_ALL	13.9333	12.7500	13.3000	53.2105
1000g2015aug_eas/ 1000g2015aug_all	4.9603	4.9766	4.4571	4.8633
OR_ExAC_	2.1815	-	3.0087	42.7604
OR_CONVERGE_	1.2602	0.979	0.8722	1.1531
ExAC_ALL	0.0015	0.0016	0.001	0.0019
ExAC_EAS	0.0209	0.0204	0.0133	0.1011
ExAC_nonpsych_ALL	0.0012	0.0013	0.0007	0.0010
ExAC_nonpsych_EAS	0.0183	0.0171	0.0098	0.1250
1000g2015aug_all	0.0042	0.0032	0.0020	0.0090
1000g2015aug_eas	0.0208	0.0159	0.0089	0.0437
Deleteriousness	9	2	1	4

**Figure 3:**
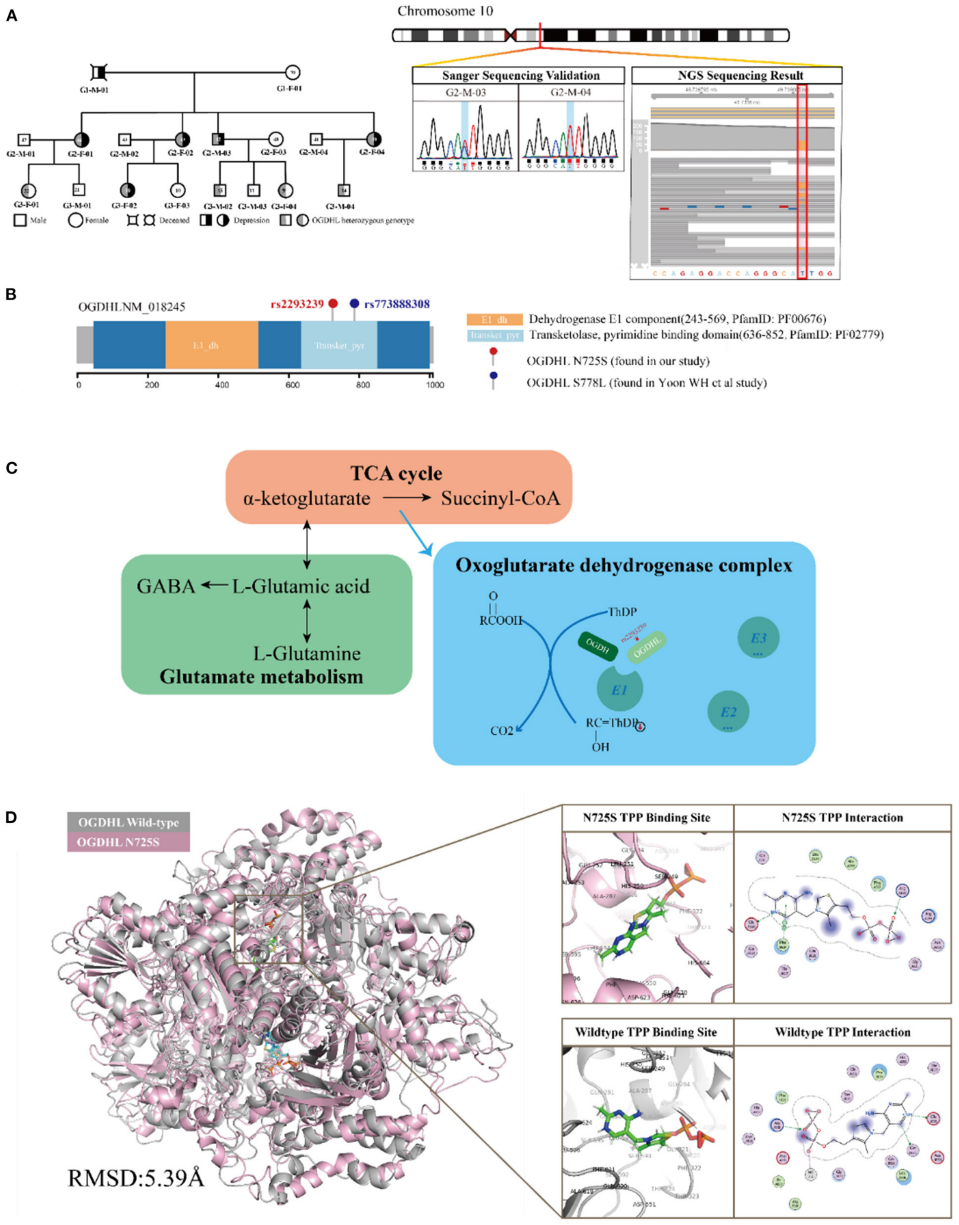
Functional annotation of the oxoglutarate dehydrogenase-like (*OGDHL*) rs2293239 variant. **(A)** *OGDHL* rs2293239 genotypes of the family members are highlighted in gray. The *OGDHL* rs2293239 genotype was validated by both whole-exome and Sanger sequencing. **(B)** The rs2293239 variant (N725S) was located at the transketolase pyrimidine binding domain of OGDHL, near the rs773888308 (S778L) variant that was identified in the study by Yoon. **(C)** The function of OGDHL in glutamate metabolism. **(D)** The conformation of OGDHL was altered by the rs2293239 mutation, and the binding affinity with cofactor thiamine pyrophosphate (TPP) was weakened by the mutation.

Of these four candidate variants, we speculated that *OGDHL* rs2293239 was most likely the driving genetic force behind depression in this family. This speculation was based on several pieces of evidence: (1) *OGDHL* rs2293239 was predicted as highly deleterious by 9/13 algorithms; (2) this variant had a slightly higher frequency in the CONVERGE Chinese depressive women cohort (14) than in the non-psychotic controls (OR = 1.2602); (3) a literature search revealed that OGDHL is related to psychological diseases such as Alzheimer's disease (53), childhood disintegrative disorder (54), and cerebral atrophy (55); and (4) OGDHL is highly expressed in all regions of the human brain according to the Tissue Atlas database (56, 57). Together, these findings suggest that *OGDHL* rs2293239 is connected with depression. The genetic status of *OGDHL* rs2293239 in the present family is illustrated in Figure 3A, and the genotypes of this allele were further confirmed by additional Sanger sequencing (Figure 3B). Of the 17 family members, all depressive members carried this variant in a heterozygous genotype. However, we must note that four healthy third-generations also carried this heterozygous mutation but had not yet exhibited depressive or other psychotic disorders (at the time of writing).

### Mutations in OGDHL May Cause Impaired Glutamate Metabolism

OGDHL is known compete with oxoglutarate dehydrogenase (OGDH) in forming the complex E1 subunit, which is essential for catalyzing the conversion of 2-oxoglutarate (α-ketoglutarate) to succinyl-coenzyme A and CO_2_ in the citric acid cycle. According to Sen et al. (58) and Bunik et al. (53, 59), OGDH and OGDHL are isozymes that are located in different organs. OGDHL is mainly located at the mitochondrial matrix in the brain, and uses TPP as a cofactor (Figure 3C).

OGDHL has two functional domains: the E1 dehydrogenase component (PfamID: PF00676) and the transketolase pyrimidine binding domain (PfamID: PF02779) (Figure 3B). The *OGDHL* rs2293239 variant that was identified in the family was located in the transketolase pyrimidine binding domain, and resulted in a non-synonymous translation from arginine to serine at position 725 (N725S). Because there are currently no OGDHL structures available in the PDB database, we constructed one using homology modeling. The OGDHL was found to work as a dimer, and cofactor TPP binding occurred at the site between two monomers. We simulated the homodimer of OGDHL coupled with TPP as well as with Ca^2+^ and Mg^2+^ (Figure 3D). The binding pattern was analyzed and the critical residues within are illustrated in Figure 3D.

To evaluate the impact of the rs2293239 mutation on protein activity, we also simulated the structure of the OGDHL N725S variant. Compared with the wild-type protein, the N725S mutation induced a 5.39 Å conformational change (Figure 3D), which caused the binding affinity of the TPP–OGDHL dimer to decrease from −12.94 kcal/mol (wild-type) to −8.75 kcal/mol (N725S variant). It was speculated that this change in OGDHL activity may eventually impair glutamate metabolism and raise glutamate concentrations in the brain. Therefore, we measured glutamate levels in the peripheral blood (because of the difficulty in obtaining cerebrospinal fluid) of the family members. The depressive family members had higher blood glutamate levels than the healthy family members (analysis of variance). This result supported our hypothesis that the rs2293239 variant may impair OGDHL function and increase intracellular glutamate.

### Endophenotype of the OGDHL Variant

**Figure 4:**
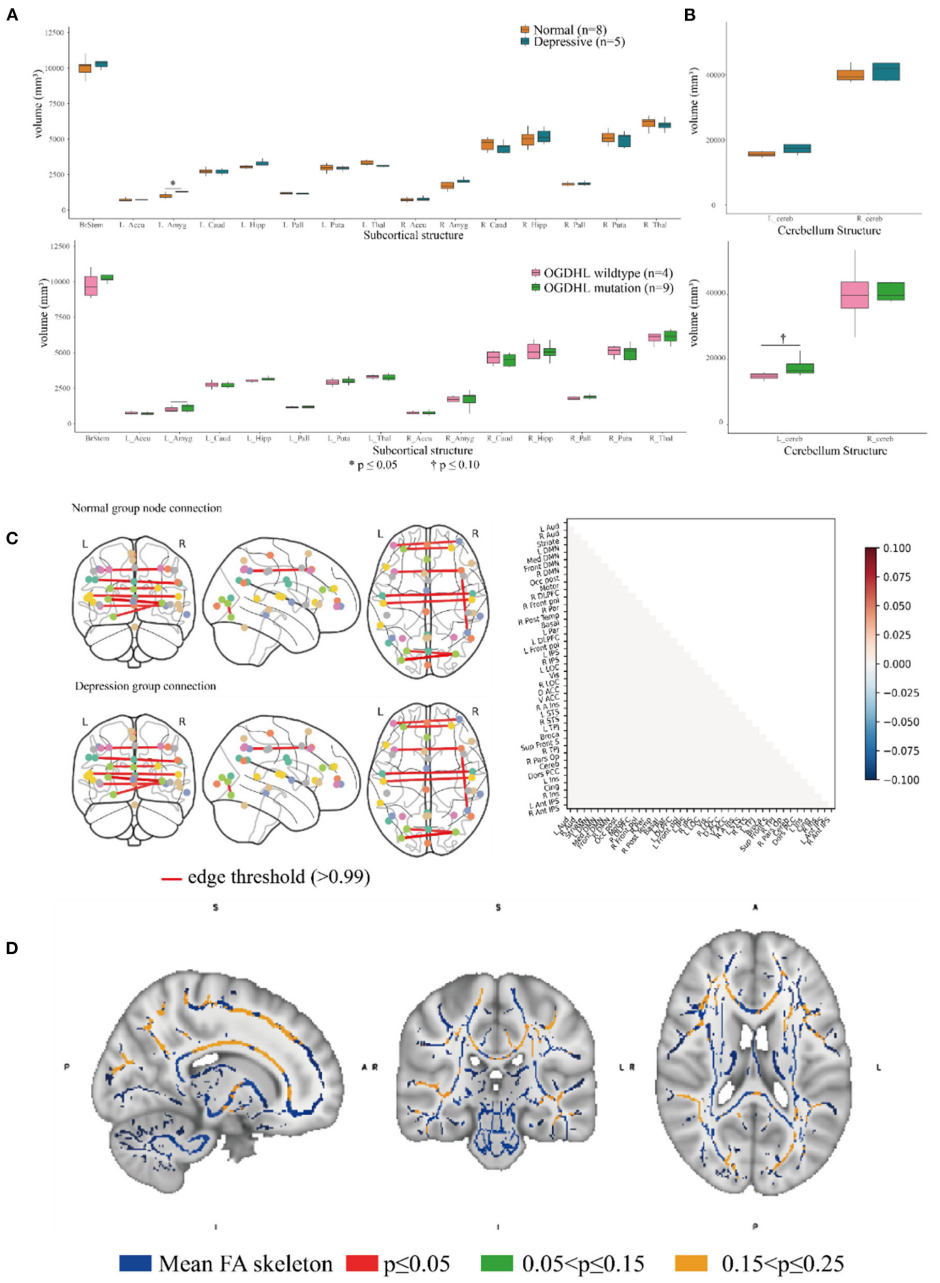
Association analysis of the brain endophenotype with the oxoglutarate dehydrogenase-like (*OGDHL*) rs2293239 variant. **(A)** Volume comparison of the subcortical brain structures. The depressive family members had a significantly enlarged left amygdala compared with the healthy family members (*t*-test, *p* = 0.0391); however, this volume difference was not significant between rs2293239 carriers and non-carriers. **(B)** The rs2293239 carriers had a comparatively larger left cerebellum compared with the non-carriers, but this difference was not significant (*t*-test, *p* = 0.07). **(C)** Comparison of brain activity between rs2293239 carriers and non-carriers based on the connectome of 39 distinct brain regions, *via* an analysis of the time-series of blood oxygen level changes determined by functional magnetic resonance imaging (MRI). **(D)** Neural tract skeleton integrity analysis based on diffusion MRI. No significant skeleton defects were detected.

Brain imaging can provide helpful information for identifying endophenotypes in the central nervous system that are caused by genetic mutations. Previously, Yoon et al. reported that the homozygous mutation S778L in *OGDHL*, which is located at a domain near rs2293239, caused severe neurodegeneration in a 13-year-old patient (55). In the current study, we performed brain imaging analyses for the family members in as much detail as possible (Figure 1). The images were evaluated in three separate analyses. First, we compared the volumes of 15 distinct subcortical structures between five depressive family members and eight healthy family members. There was a significant (*t*-test, *p* = 0.03908) volume increase in the left amygdala in the depressive family members (Figure 4A). This result was consolidated by the permutation test, which randomized the depressive and healthy members 10,000 times (*p* < 0.05) for the differential volume analysis. This finding agreed with the results of previous studies, that MDD patients have comparatively larger amygdalae (60–62). However, there were no significant volume differences in the left amygdala between nine rs2293239 carriers and four family members with wild-type *OGDHL*. Interestingly, a slight but non-significant volume increase (*t*-test, *p* = 0.07) in the left cerebellum was also noted in the nine rs2293239 carriers (Figure 4B). In both comparative analyses, no changes in the shape of subcortical or cerebellar structures were observed.

Second, we examined the integrity of neural tract skeletons by analyzing dMRI images using a TBSS method. MRI data did not reveal any significant differences among the five patients diagnosed with depression, including structural, functional, and DTI alterations. No significant skeleton defects or structural differences were identified between either the rs2293239 carriers or the depressive family members and the controls (Figure 4C). Finally, we attempted to evaluate changes in brain activity. However, in our fMRI analysis of a time-series of blood oxygen level changes, we did not detect any significant differences in activity in the connections between 39 distinct brain regions (Figure 4D). Therefore, it was considered that the *OGDHL* rs2293239 variant was unlikely to induce depression by interfering with cerebral cortex activity in this family.

## Discussion

### OGDHL Variant May Be One of the Major Genetic Factors of Depression Onset

Depressive disorders are heterogeneous in their pathophysiology. To date, identifying the major pathological factors for depression onset in each individual has been something of a “mission impossible,” and the development of precise therapeutic regimens for individuals has also been challenging. Genetic mutations are estimated to account for approximately one-third of all depressive disorders (5, 6, 63, 64). Because genetic factors are inherited from generation to generation, they are the most likely common pathological factors to be identified for depressive disorders. Many studies of twin, pedigree, and case–control cohorts have been performed worldwide, and have identified a variety of depression susceptibility genes, such as *SLC6A4, APOE, DRD4, GNB3, HTR1A, MTHFR*, and *SLC6A3* (11). Unfortunately, however, many of these susceptibility genes have not been be repeated in larger populations or in other cohorts (16). GWAS studies have complemented the population gap, but the common somatic variants identified by these studies mostly have low penetrance for depressive disorders. The poor interpretation and repetition of GWAS results have also meant that such findings have been questioned (11, 16). Notably, using low-coverage whole-genome sequencing, a 2015 case–control study of 5,303 Chinese women with recurrent MDD and 5,337 controls identified two risk loci, at noncoding regions in *SIRT1* and *LHPP* (14). A similar result was obtained in a large Japanese cohort study, which identified that the *SIRT1* rs10997875 variant was associated with MDD (65). Furthermore, a mouse study suggested that hippocampal SIRT1 signaling can mediate depression-like behaviors (66), and a clinical trial also observed significantly reduced peripheral blood *SIRT1* mRNA in depressive patients compared with healthy controls (67). Together, these findings indicate that *SIRT1* is highly susceptible to depression and may be a good therapy target. However, opposing findings have also been reported; in one study, mice with global *SIRT1* overexpression had elevated anxiety and increased susceptibility to depression (68). In the current study, we also evaluated *SIRT1* variants and their penetrance in the family. Of three distinct susceptible variants (an exonic variant, rs2273773, and two novel intronic variants) that were identified in and near *SIRT1*, none showed a significant association with depression. In fact, none of the common variants identified in this family were associated with depression.

Instead of seeking common somatic variants, we discovered rare germline variants with high penetrance for depression in the present study. *Via* a series of bioinformatic analyses, we mined four potential driver variants: *OGDHL* rs2293239, *TBX1* rs41298838, *IL16* rs201457933, and *FBXO15* rs79499419 (Table 3). These variants satisfied multiple criteria: a strong association with depression, low frequency in the population (less than or nearly equal to the depression incidence rate in Chinese), high deleteriousness, and fit a Mendelian inheritance model.

T-box transcription factor (TBX1) is a probable transcriptional regulator that is involved in development. TBX1 is responsible for most of the physical malformations present in 22q11.2 deletion syndrome, or DiGeorge syndrome, which is a congenital disease that has been implicated in various behavioral abnormalities including schizophrenia and other neuropsychiatric/behavioral disorders (69–71). A recent study also reported that TBX1 in the mouse hippocampus might be linked to monosaccharides–D-ribose-induced depressive-like behavior (72). In the present study, the rare variant rs41298838 was predicted to be highly deleterious to TBX1 function, which made it a potential driving variant for familial depression. However, a close review of this variant in the general population revealed that it has a comparatively high allele frequency (more than 10%) in non-psychotic Asians, according to the ExAC database. In addition, WES analysis and karyotyping did not identify 22q11.2 deletions or other chromosomal arrangements in the family members. These data challenge the idea that TBX1 rs41298838 might be a good candidate for familial depression. Interleukin (IL)-16 is the ligand of cluster of differentiation (CD)4, and can stimulate a migratory response in CD4^+^ lymphocytes, monocytes, and eosinophils. Several studies have used IL-6, as well as other cytokines, as indicators for monitoring the immune response in the treatment and pathogenesis of affective disorders (73, 74). However, no substantial evidence indicates that IL-6 is directly involved in the development of depressive or other affective disorders. F-box only protein 15 (FBXO15) is the substrate-recognition component of the E3 ubiquitin ligase complex, which is involved in ubiquitin–proteasome-mediated protein degradation. To date, knowledge of the function of FBXO15 remains very limited, and its association with affective disorders or nerve diseases has not yet been well studied. Nonetheless, considering the uncertain relevance of IL-16 and FBXO15 for depressive disorders, we did not prioritize them in the functional investigation.

Compared with TBX1, IL-16, and FBXO15, we found that OGDHL was strongly associated with depressive disorders in a range of different aspects. First, a series of bioinformatic analyses indicated that the *OGDHL* rs2293239 variant had a strong association with depression onset in the present family. The rs2293239 variant is a very rare mutation in global population (about 0.1%); it is comparatively more frequent in Asian populations (about 2%), and its allele frequency is nearly equal to the incidence rate of depression in Asian populations (75). A previous case–control cohort study (14) reported that the rs2293239 variant occurs more frequently in Chinese depressive women than in nonpsychotic controls (OR = 1.2602). These data suggest that rs2293239 may not be a common driving genetic force for all cases of depressive disorders. However, the rs2293239 mutation in the transketolase pyrimidine binding domain is likely very deleterious, as predicted by nine algorithms in the present study. *Via* a structure–activity relationship analysis, we revealed that the mutation may cause an important conformational change (about 5.39 Å) in the protein, which would decrease the binding affinity of *OGDHL* dimer with its cofactor TPP. *OGDHL*, which competes with the dominant player *OGDH*, is the rate-limiting component of the oxoglutarate dehydrogenase complex in glutamate metabolism. Inhibition of *OGDH*(L) results in a two- to three-fold increase in glutamate in neuronal cells (76). Glutamate is a major neurotransmitter in more than 80% of neurons (77), and abnormal glutamate production in neurons and glial cells can cause severe depressive symptoms (78). Thus, we measured serum glutamate levels in the depressive family members. An additional literature search also supported the relationship between *OGDHL* and psychological diseases. For example, an early study proposed that *OGDHL* might be linked to neurotransmitter synthesis and Alzheimer's disease (53). Furthermore, aberrant *OGDHL* copy number may be associated with childhood disintegrative disorder (54). In particular, the homozygous mutation rs773888308 (S778L) of *OGDHL*, which is a mutation at the transketolase pyrimidine binding domain near rs2293239 (N725S), was found to cause a severely hypoplastic corpus callosum and abnormal cerebellum in a 13-year-old patient (55). This previous work strongly supports our speculation that the rs2293239 mutation likely impairs *OGDHL* catalytic activity, thus resulting in increased intracellular glutamate and eventually inducing depression.

### OGDHL Variant May Cause Brain Structural Changes

In 2017, Yoon et al. attributed the homozygous *OGDHL* mutation rs773888308 (S778L) to severe neurodegeneration and encephalatrophy in a girl (55). In the present study, we also measured brain structural changes using MRI techniques, and revealed that depressive family members had a significantly enlarged left amygdala compared with healthy family members. This finding agreed with the results of previous cohort studies, that MDD patients have a larger amygdala and smaller anterior cingulate compared with normal controls, with medium to large effect sizes (60–62). Notably, the endophenotype change in the amygdala was not observed in all carriers of *OGDHL* rs2293239 in the family; three third-generation young variant carriers did not exhibit changes in either the volume or the shape of the amygdala. MDD patients have been reported to have comparatively enlarged amygdalae (79), however this phenomenon may be related to long-term antidepressant use given that the enlargement is less reliably observed in non-medicated patients with MDD (80). It has been suggested that anti-depressive pharmacotherapy associated induction of brain-derived neurotrophic factor and other neuroprotective factors may have a volume-augmenting effect on the amygdala (81). However, these three variant carriers had a comparatively, although non-significantly (*p* = 0.07), enlarged left cerebellum compared with the non-carriers. In addition, no neural tract defects were identified in the depressive family members via dMRI examination. Moreover, a further case–control comparative analysis of the connectome of different cerebral cortex nodes (regions) did not identify any significant changes in cerebral activity, as indicated by a time-series of blood oxygen levels. It is therefore reasonable to suspect that the changes in cerebral substructures caused by *OGDHL* rs2293239 may account for the depressive disorders of this family. However, because this genetic alteration was heterozygotic, it may be that *OGDHL* protein function is only partially defective, and that brain structural changes progress so slowly that depressive symptoms are not apparent in teenagers. It will be necessary to perform animal experiments in the future to validate this hypothesis.

**Figure 5:**
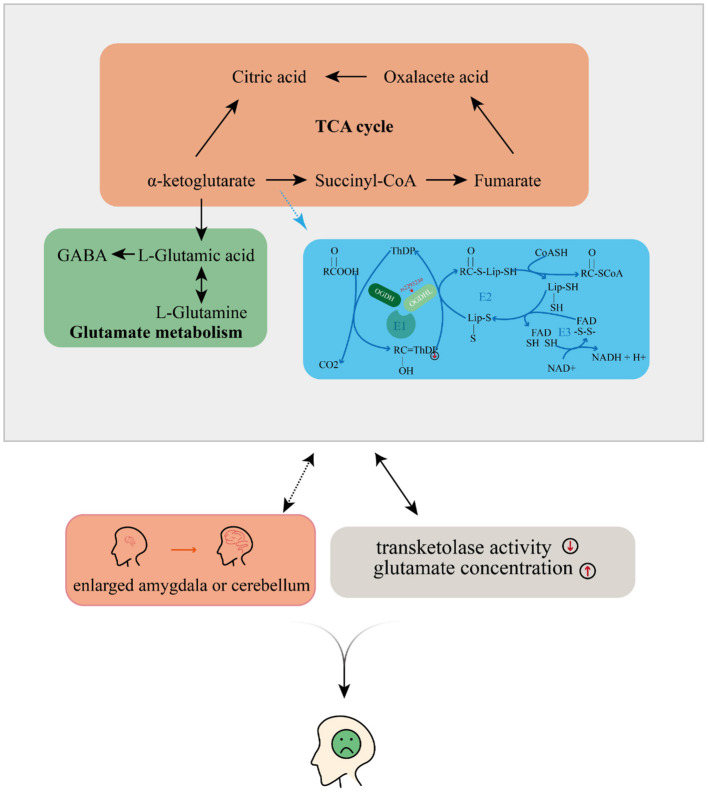
Possible mechanism by which depressive disorders may be induced by the oxoglutarate dehydrogenase-like (*OGDHL*) mutation.

Taking all of our results and the evidence from previous literature together, we propose that *OGDHL* rs2293239 is likely one of the major genetic driving forces of depression onset in the present family, via the following mechanism (Figure 5). The rs2293239 variant decreases the binding affinity of the *OGDHL* dimer to its crucial catalytic cofactor TPP, thus weakening its transketolase activity. This alters the glutamate metabolism balance in cells, resulting in increased glutamate concentrations in the brain. As a result of long-term glutamate imbalance, brain structures may change, such as enlargement of the amygdala or cerebellum; furthermore, as a result of glutamate unbalance and brain structural changes, depressive symptoms appear. This hypothesis is partially supported by a recent study that demonstrated that glutamate concentrations in the amygdala may be related to depressive moods (82).

Nevertheless, there are several uncertainties in the aforementioned hypothesis. The impact of the rs2293239 variant on glutamate metabolism has not yet been experimentally validated, although both computational simulations in the present study and previous work (76) suggest that an inhibition or defect of *OGDHL* can cause aberrant glutamate metabolism. In the family in the present study, the glutamate concentrations in the amygdala, cerebellum, and other brain structures are unknown because of the difficulty in obtaining cerebrospinal fluid. However, the relatively high peripheral serum glutamate concentration in this family partially supports our hypothesis because rs2293239 is a germline mutation, and should therefore take effect all over the body. Furthermore, causality has not yet been confirmed between brain structural changes and this variant. Although a study by Yoon et al. (55) suggested a strong association between an *OGDHL* homozygotic mutation and neurodegeneration, the penetrance of heterozygotic mutations for brain structural changes requires further verification by animal experiments. Notably, the rs2293239 variant was the most likely driving mutation mined from hundreds of thousands of germlines SNVs, Indels, and CNVs *via* a series of conditions. It is not the only genetic factor in familial depression, but it might be the most dominant one. Indeed, the relatively weak association (OR = 1.2602) of this variant with Chinese depressive women (14) suggests that it may not be a common driving genetic force for all cases of depressive disorders.

## Conclusion

We identified a novel rare variant of *OGDHL*, rs2293239, that may serve as the driving force for depression onset in a Chinese pedigree. Combining multi-omics integration and multimodal imaging analysis, or imaging genetics, we revealed the possible mechanism underlying familial depression in this pedigree. This work enhances the current molecular understanding of the complex pathogenesis of depressive disorders. Furthermore, it provides new options for prioritizing better clinical therapeutic regimens and suggests a new protein target for the design of highly specific drugs to treat depressive disorders.

## Data Availability Statement

The datasets generated and analyzed during the present study are available from the corresponding author upon reasonable request.

## Ethics Statement

The studies involving human participants were reviewed and approved by Clinical Research Ethics Committee of the Xiamen Xianyue Hospital, Fujian, China. The patients/participants provided their written informed consent to participate in this study.

## Author Contributions

CZ, ZZ, and HT conceived and designed research. ZP, ZZ, and WW collected data and conducted research. TF, ZL, XL, GD, and GH analyzed and interpreted data. WW and CZ wrote the initial paper. HT and GC revised the paper. WW and GC had primary responsibility for final content. All authors read and approved the final manuscript.

## Funding

This study was supported by the National Natural Science Foundation of China (81871018 to WW).

## Conflict of Interest

The authors declare that the research was conducted in the absence of any commercial or financial relationships that could be construed as a potential conflict of interest.

## Publisher's Note

All claims expressed in this article are solely those of the authors and do not necessarily represent those of their affiliated organizations, or those of the publisher, the editors and the reviewers. Any product that may be evaluated in this article, or claim that may be made by its manufacturer, is not guaranteed or endorsed by the publisher.

## Abbreviations

OGDHL: Oxoglutarate Dehydrogenase L
SLC6A4: Sodium-and-chloride-dependent solute carrier family 6 gene number 4
5-HTTP: 5-Hydroxy Tryptamine Transporter Polymorphism
APOE: Apolipoprotein E
DRD4: Dopamine D4 receptor gene
GNB3: G protein subunit beta 3
HTR1A: 5-Hydroxytryptamine receptor 1A
MTHFR: Methylenetetrahydrofolate reductase
BICC1: BicC family RNA binding protein 1
SIRT1: Sirtuin 1
dbNSFP: database for Nonsynonymous SNPs&#8217
functional predictions
SIFT: Sorts intolerant from tolerant
PolyPhen2 HDIV: Polymorphism Phenotyping v2 HumDiv
PolyPhen2 HVAR: Polymorphism phenotyping v2 HumVar
LRT: Likelihood ratio test
FATHMM: Functional analysis through hidden Markov models
PROVEAN: Protein variation effect analyzer
MetaSVM: Meta Support vector machine
MetaLR: Meta Logistic regression
GERP++: Genome evolutionary rate profiling ++
PhyloP: Phylogentic p-values
ff99SB-ILDN: Force field99
MOE: Molecular operating environment
LRRK2: Leucine rich repeat kinase 2
MUC19: Mucin 19
CNTN1: Contactin 1
TDRD9: Tudor domain containing 9
KIF26A: Kinesin family member 26A
C14orf180: Chromosome 14 open reading frame 180
TMEM179: Transmembrane protein 179
LRRK2: Leucine rich repeat kinase 2
CHSY1: Chondroitin sulfate synthase 1
SELENOS: Selenoprotein S
SNRPA1: Small nuclear ribonucleoprotein polypeptide A1
PCSK6: Proprotein convertase subtilisin/kexin type 6
TM2D3: TM2 domain containing 3
TARS3: Threonyl-TRNA synthetase 3
OR4F6: Olfactory receptor family 4 subfamily F member 6
OR4F15: Olfactory receptor family 4 subfamily F member 15
OR4F4: Olfactory receptor family 4 subfamily F member 4
TBX1: T-Box transcription factor 1
IL16: Interleukin 16
FBXO15: F-Box protein 15
LHPP: Phospholysine phosphohistidine inorganic pyrophosphate phosphatase
ExAC: Exome aggregation consortium
WES: Whole exome sequencing.
